# Are Aortic Stent Grafts Safe in Pregnancy?

**DOI:** 10.1155/2015/190878

**Published:** 2015-07-01

**Authors:** Nader Khandanpour, Tapan A. Mehta, M. Adiseshiah, Felicity J. Meyer

**Affiliations:** ^1^St Georges NHS, London SW17 0QT, UK; ^2^Bedford Hospital, Kempston Road, Bedford MK42 9DJ, UK; ^3^University College London Hospital, 235 Euston Road, London NW1 2BU, UK; ^4^Norfolk and Norwich Vascular Unit (NANVU), Norfolk & Norwich NHS Trust, Colney Lane, Norwich NR4 7UY, UK

## Abstract

Aortic stent grafts are increasingly used to treat aortic aneurysms and also other aortic pathologies. The safety of aortic stent grafts in pregnancy has never been studied or reported. We report on two cases of aortic stent grafts in pregnant women and discuss the effect of pregnancy on these aortic stent grafts.

## 1. Introduction

In recent years there has been an exponential rise in the use of aortic stent grafts. Besides aneurysm repair, aortic stent grafts are also useful in acute and chronic aortic dissections, traumatic transections of the aorta, and selected cases of coarctation of the aorta [1]. A detailed literature review failed to show limited information about the use and safety of aortic stent grafts in pregnant women. We describe two cases of women with aortic stent grafts who became pregnant and discuss the effect of pregnancy on these stent grafts.

## 2. Case 1

**Figure 1:**
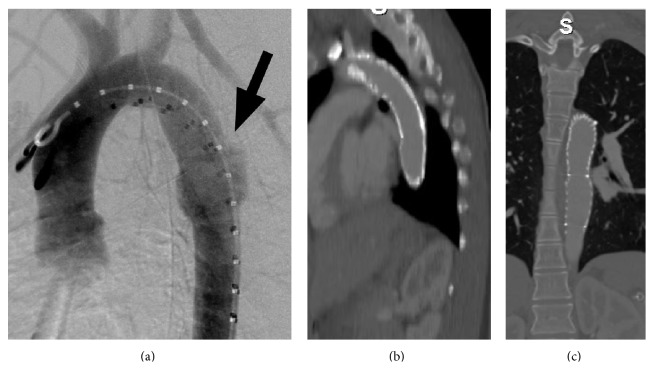
(a) Transection of descending thoracic aorta in 19-year-old multitrauma victim. (b) The stent graft lies in satisfactory position and patent-sagittal view. (c) The stent graft lies in satisfactory position and patent-coronal view.

A 19-year-old woman was involved in a major road traffic accident which resulted in multiple injuries including a cervical spine fracture, liver and spleen injuries, pelvic fracture, and a traumatic transection of the descending thoracic aorta (Figure 1(a)). The aortic transection was treated with an emergency aortic stent graft and she also underwent a laparotomy with splenectomy. After a long stay in hospital she made an uneventful recovery. The following year she became pregnant and presented at 17 weeks of pregnancy with abdominal and chest pain with some shortness of breath. A transthoracic echo revealed that the aortic stent graft had collapsed. This was treated with balloon angioplasty and a further Palmaz stent to support the stent graft (Figures 1(b) and 1(c); Table 1). She underwent an elective Caesarean section at 39 weeks, which was otherwise uneventful. She is now progressing well in her second pregnancy.

## 3. Case 2

**Figure 2:**
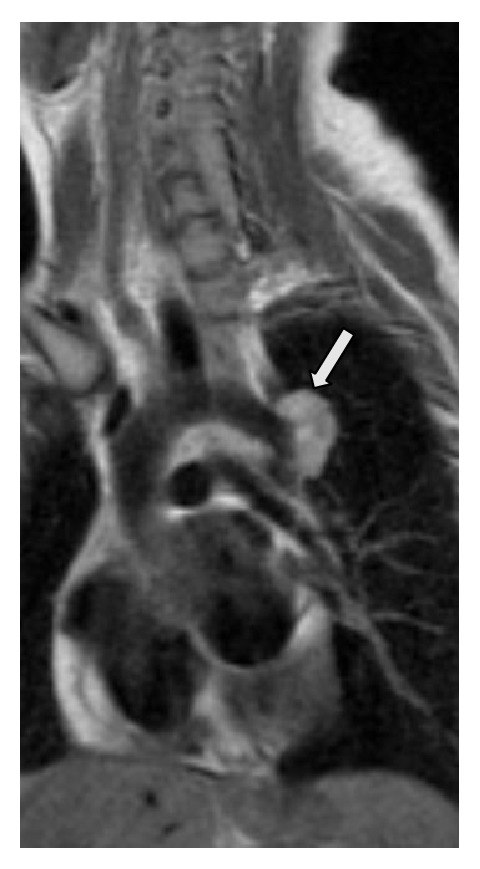
The pseudoaneurysm with thrombus at the Dacron patch repair site found during a routine antenatal surveillance.

**Figure 3:**
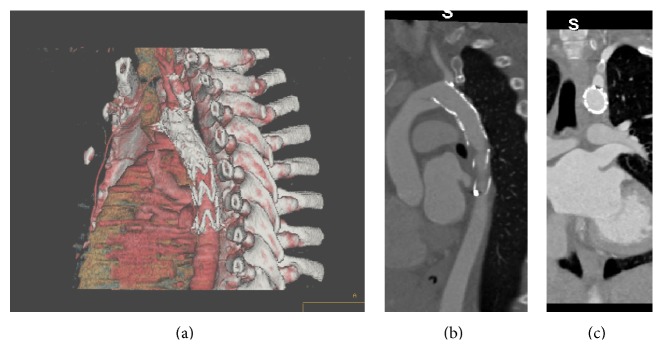
(a) Thoracic stent graft partially covering the origin of the left subclavian artery-3D reconstruction. (b) The stent graft partially covers the origin of the left subclavian artery. (c) The stent graft partially covers the origin of the left subclavian artery.

A 28-year-old woman was first seen in the antenatal clinic at 20 weeks of gestation. She had a history of coarctation of the aorta which was treated shortly after her birth with resection and end-to-end anastomosis. At age six, a Dacron patch repair was performed for residual stenosis. The patient was asymptomatic and on routine antenatal surveillance she was found to have a pseudoaneurysm with thrombus at the Dacron patch repair site (Figure 2). This was treated conservatively with anticoagulation and strict blood pressure control and an elective Caesarean section at 29 weeks. In the week following the Caesarean section the pseudoaneurysm was covered endovascularly with an aortic stent graft. The proximal end of this stent graft partially covered the origin of the left subclavian artery (Figures 3(a), 3(b), and 3(c)) but this was asymptomatic at the time and was therefore ignored. A year later the patient developed some left arm claudication, which was managed expectantly and resolved spontaneously in a few months. Three years later the patient became pregnant again and during the third trimester of this pregnancy she developed some transient trapezius muscle ischaemia. This was suspected to be due to altered circulatory haemodynamics related to the partially covered origin of the left subclavian artery (Table 1). This too was managed expectantly and resolved spontaneously. The patient was delivered by Caesarean section at 36 weeks without incident and she has subsequently remained asymptomatic.

## 4. Discussion

Parodi et al. first used an aortic stent graft to treat an aneurysm in 1991 [2]. There are some salient differences between stents and stent grafts. In their usual design, stent grafts have a main body which is covered and made of fabric reinforced by metal struts and/or wire mesh. This part is used to exclude parts of the aortic lumen from the main channel of blood. The stent graft is held in place at the proximal and distal end by self-expanding stents which grip the native aortic wall. Changes in the aortic wall can affect the position of the aortic stent graft. In addition to treating aneurysms of the aorta, aortic stent grafts have other uses and in young adults these commonly include traumatic transections of the aorta (as in case 1) and exclude other aortic leaks (as in the pseudoaneurysm in case 2).

Pregnancy poses significant physiological alterations to the cardiovascular system. These are mainly due to the metabolic demands brought on by the foetus, placenta, and uterus and the increasing levels of pregnancy hormones, that is, progesterone and oestrogen. The maternal blood volume increases progressively from 6 to 8 weeks of gestation and reaches a maximum at 32–34 weeks. The plasma volume increases by 40–50%, which is greater than the red cell mass increase of 20–30%. There is a steady increase in cardiac output in pregnancy. This is due to a combination of a more rapid heart rate (15%) and an increase in stroke volume (35%). Pregnancy hormones progesterone and relaxin have a relaxing effect on smooth muscles including vascular smooth muscle cells. There is a steady reduction in systemic vascular resistance during pregnancy [3, 4]. These changes have a significant impact on the aortic wall during pregnancy. In our first case the aortic stent graft collapsed in mid pregnancy. In the second case the aortic stent graft was inserted in early puerperium before the vascular changes of pregnancy could revert back to normal. This may have caused a subtle migration of the stent graft a year later causing arm claudication. Stent graft migration and subsequent endoleaks have been described after aortic aneurysm repair and occur as the excluded aneurysm shrinks in size. This is the first report of adverse alterations to aortic stent grafts during and after pregnancy.

Another issue of concern is the risk of radiation exposure during pregnancy from various imaging modalities. The International Atomic Energy Association (IAEA) has issued guidelines on pregnancy and radiation protection in diagnostic radiology. These are based on the International Commission on Radiological Protection (ICRP) publication 84 [5]. It is unlikely that radiation from diagnostic radiological examinations will result in any deleterious effects on the baby, but the possibility of radiation-induced effect cannot be entirely ruled out. The mean and maximum foetal radiation doses from computed tomography (CT) examination of the chest are 0.06 and 0.96 mGy. The foetal radiation exposure during thoracic angiography has only been studied as extrapolation data from cardiac catheterization. The foetal radiation exposure mainly occurs from scattered radiation within the patient. Some methods to minimize foetal radiation exposure include restricting X-ray beam size, limiting exposure time, and altering the direction of the primary beam. The early involvement of a knowledgeable medical physicist is desirable.

The use of aortic stent grafts requires caution in women of child bearing age. Such women should be counselled regarding unplanned pregnancies and require close surveillance during and after pregnancy.

## Figures and Tables

**Table 1:** Summary of the details of the procedures and outcomes.

	Number of radiology procedures/scans during pregnancy	Type of graft	Follow-up
Patient 1	1 catheter angiogram	Gore Tag thoracic device put in initially, then ballooned, and Palmazed	2 years stable

Patient 2	0	Medtronic Talent	2 years stable
